# Omnidirectional optical attractor in structured gap-surface plasmon waveguide

**DOI:** 10.1038/srep23514

**Published:** 2016-03-22

**Authors:** Chong Sheng, Hui Liu, Shining Zhu, Dentcho A. Genov

**Affiliations:** 1 National Laboratory of Solid State Microstructures & School of Physics, Collaborative Innovation Center of Advanced Microstructures, National Center of Microstructures and Quantum Manipulation, Nanjing University, Nanjing 210093, China; 2 College of Engineering and Science, Louisiana Tech University, Ruston, Louisiana 71270, USA

## Abstract

An optical attractor based on a simple and easy to fabricate structured metal-dielectric-metal (SMDM) waveguide is proposed. The structured waveguide has a variable thickness in the vicinity of an embedded microsphere and allow for adiabatic nano-focusing of gap-surface plasmon polaritons (GSPPs). We show that the proposed system acts as an omnidirectional absorber across a broad spectral range. The geometrical optics approximation is used to provide a description of the ray trajectories in the system and identify the singularity of the deflection angle at the photon sphere. The analytical theory is validated by full-wave numerical simulations demonstrating adiabatic, deep sub-wavelength focusing of GSPPs and high local field enhancement. The proposed structured waveguide is an ideal candidate for the demonstration of reflection free omnidirectional absorption of GSPP in the optical and infrared frequency ranges.

The possibility to capture and focus light in deep sub-wavelength spatial domains and thus enhance the interaction between light and matter, has recently attracted substantial interest from the optical community. Owing to ongoing advances in optics and nanotechnology, various plasmonic structures such as tapered waveguides^1^,^2^, metallic tips^3^, wedge and V-grooves^4^,^5^,^6^, has been shown to guide and concentrate surface plasmon polaritons (SPPs), i.e. electromagnetic waves that travel along the interface between metal and dielectric, within nanometer size domains. In such systems the propagation of SPPs could be adiabatically slowed down until the surface waves virtually stop and are transformed into localized modes at the tip of these tapered structures. As a result of the energy localization, enhanced light-matter interaction can be manifested including enhanced Raman scattering^7^,^8^, enhanced spontaneous emission of radiation and nano-lasing^9^,^10^,^11^ , bio-sensing^12^ and nano-resolution optical imaging^13^.

In recent years the transformation optics^14^,^15^,^16^,^17^ approach, which provides for an unprecedented control over light through design of inhomogeneous magneto-dielectric materials, has enabled the manifestation of exciting effects such as electromagnetic invisibility^18^,^19^,^20^,^21^,^22^,^23^,^24^,^25^,^26^,^27^,^28^,^29^,^30^,^31^,^32^, illusion optics^33^,^34^,^35^,^36^, imaging with super resolution^37^,^38^, bending waveguide without losses^39^,^40^, and even mimicking general relativity phenomenon^41^,^42^,^43^,^44^,^45^. The transformation optics has also been applied to design plasmonic elements with structural “singularities”, such as touching nano-cylinders and nano-particles with crescent shapes, which can efficiently harvest light by concentrating it energy into nano-metric volumes over a broad frequency spectrum^46^,^47^,^48^,^49^. In these systems, as the electromagnetic waves propagate toward the structure’s “singularity” their wavelength shortens and group velocity decreases similarly to what happens in sharp metallic tips and grooves. Alternatively, recent research^50^,^51^,^52^,^53^,^54^,^55^ has shown that light can be trapped in a process that mimics gravity. Two main approaches for recreating optical attractors or “black holes” in the lab have been proposed namely through use of either inhomogeneous composite media with position dependent refractive index^51^,^55^ or structured waveguides and surfaces^54^. The main issue faced by these approaches is the need to manufacture complex materials and structures with strictly-specified position dependent and exceedingly large effective refractive indexes.

In this work, we propose a simple and easy to fabricate structured metal-dielectric-metal (SMDM) that addresses the two requirements for recreating an optical attractor in the lab, namely it naturally establishes a precise inverse square law refractive index profile which also achieves exceedingly high values at a singular point. This is accomplished through a synergy between the structured waveguide^51^,^55^ and gap surface plasmon^54^,^56^ approaches, by embedding a microsphere within a metal-dielectric-metal (MDM) waveguide to provide a well-defined variable waveguide thickness and hence modulation of the effective refractive index of the GSPP modes supported by the structure. Using the geometrical optics approach we have developed analytical theory to describe the propagation of the SPPs inside the system. The theory predicts formation of a photon sphere for in-falling rays with an impact factor below a critical value which can be easily tuned changing the diameter of the sphere and frequency of excitation. The in-falling light rays are shown to asymptotically approach a central singularity and are adiabatically absorbed inside the optical attractor. Finally, the analytical theory is validated by full-wave numerical simulations, demonstrating the critical characteristics of the proposed optical attractor, including field localization, omnidirectional and spectrally broad absorption of the impinging GSPP waves. Possible applications in devices performing energy harvesting or facilitating enhancement of optical nonlinearities are discussed.

## Surface Plasmon Polaritions in structured metal-dielectric-metal waveguides

**Figure 1:**
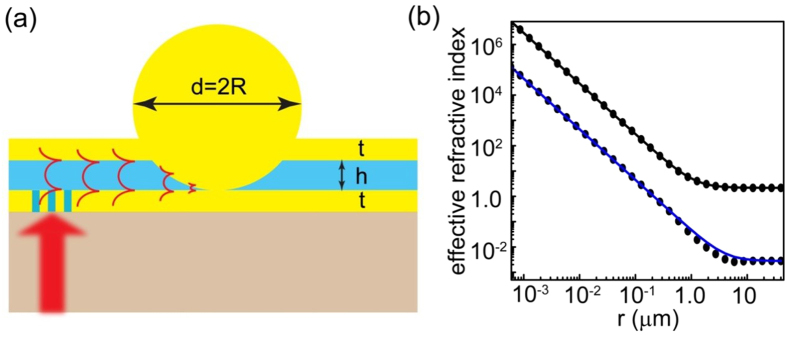
(**a**) Basic schematics of the structured metal-dielectric-metal (SMDM) waveguide. The gap-surface plasmon polarition (GSPP) modes can be excited using a grating and compressed and captured when approaching an inserted in the waveguide silver microsphere. (**b**) The effective refractive index of the symmetric GSPP modes is calculated as function of the distance to the contact point between the microsphere and bottom metal layer. The black and blue lines correspond to the real and imaginary parts of effective index calculated using the approximated result Eq. (2), while the dots are the exact values according to the GSPP dispersion relationship Eq. (1). In the calculations we have used *λ* = 0.785 *μm*, *h* = 300 *nm*, *R* = 40 *μm*.

In this work we study an optical attractor manifested for gap- surface plasmon polaritons (GSPP) in a microstructure comprised of a metal microsphere embedded within a metal-dielectric-metal (MDM) waveguide. A general schematic of the system configuration is shown in Fig. 1. The envisioned system involves a process where (i) metal (silver) microsphere is positioned on a planar silver layer, (ii) dielectric Si_3_N_4_ film with 300 nm thickness is then deposited and (iii) capped by a second thin silver film, forming the structured metal-dielectric-metal (SMDM) waveguide. The embedded microsphere provides a variable thickness for the dielectric layer pertaining to the waveguide and thus will modify the properties of its optical modes. Specifically, the SMDM waveguide supports symmetric and anti-symmetric gap- surface plasmon polariton (GSPPs) modes^57^ with effective refractive indexes that strongly depend on the waveguide thickness *h*. This dependence is used to generate the required refractive index profile for the proposed in this work optical attractor. In what follows we consider only the symmetric SPP mode of the waveguide which has a dispersion relationship given as


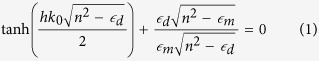


where *n* is the complex valued effective refractive index of the SPPs, *ε*_*m*(*d*)_ are the permittivities of the metal(dielectric) components, and *k*_0_* = ω/c* is the wave vector in free space. The permittivity of the metallic (silver) components is described using the Drude model 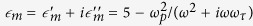, with plasma frequency *ω*_*p*_ = 9.1 *eV* and relaxation rate *ω*_*τ*_ = 0.021 *eV*^58^, while the dielectric layer is assumed Si_3_N_4_ with *ε*_*d*_ = 4.

As stated above the microsphere modifies the SMDM thickness according to the surface profile *h*(*r*) = *R* − (*R*^2^ − *r*^2^)^1/2^ (for *r* ≤ *R*), where *R* is the sphere radius and *r* is the radial distance from the point of contact. Accordingly, a position dependent GSPP refractive index is expected. While the SPP dispersion Eq. 1 cannot be solved explicitly for the effective refractive index of the waveguide mode, an excellent approximation can be obtained taking into account that the argument of the hyperbolic tangent function is rather small in the case of GSPPs. Specifically, keeping terms of first order in *hk*_0_ ≪ 1 and *r*/*R* ≪ 1 and using Eq. 1 we obtain:


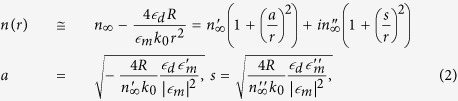


where 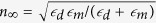 is the asymptotic value of the GSPP refractive index for *h* → ∞ . A comparison between the GSPP effective refractive index calculated using the dispersion relationship Eq. (1) and the explicit result given by Eq. (2) is shown in Fig. 1(b). Both results are in good correspondence showing that exceedingly high effective refractive indexes for GSPPs can be achieved using the proposed SMDM waveguide. The central symmetry of the system and the inverse square dependence of the effective index on the radial distance provide all the ingredients for developing an optical attractor.

### Ray trajectories, turning points and photon sphere

**Figure 2:**
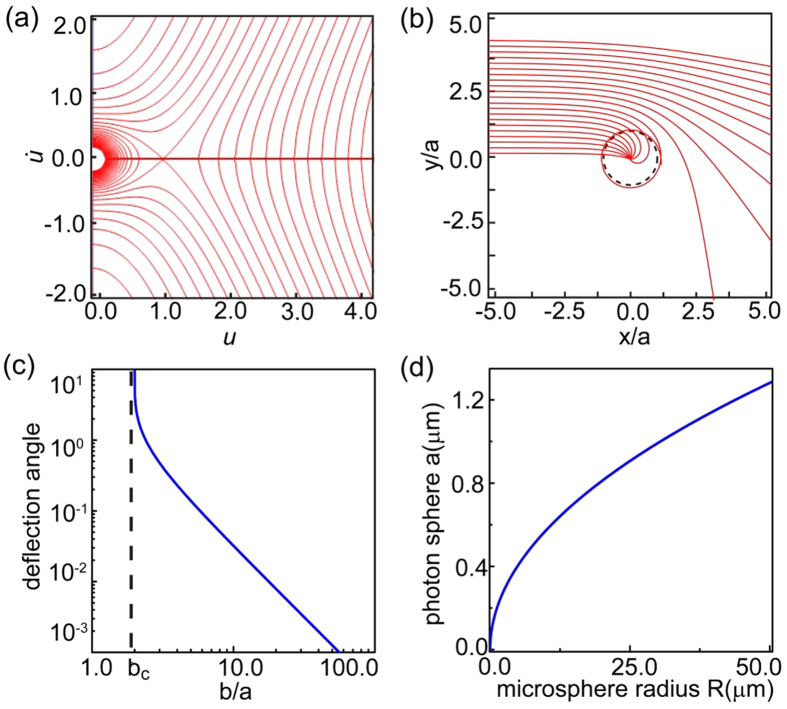
(**a**) Phase space diagram of the SPP ray trajectories in the SMDM waveguide. (**b**) A set of SPPs trajectories in real space for various impact parameters *b*. For impact parameters larger than critical value *b* > *b*_c_ = 2*a*, the SPPs are scattered back into infinity, while for impact parameters below the critical all rays are captured by the attractor. In the figure the photon sphere (*r*≅*a*) is identified with dashed line. (**c**) The total deflection angle for in-falling rays with *b*≥*b*_c_ calculated using Eq. (6). (**d**) The relationship between the SMDM photon sphere radius *a* and the radius of the sliver microsphere sphere radius *R*. In all calculations we have used *λ* = 0.785 *μm*, and *h* = 300 *nm*.

Here, we show that the proposed SMDM waveguide constitutes an omnidirectional and spectrally broad attractor of gap-surface plasmon polaritons (GSPPs). Under the geometrical optics approximation, the propagation of the GSPPs in the centrally symmetric effective index Eq. (2) (using the real part of the refractive index only), can be described by the Lagrangian


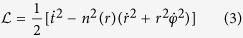


where the derivatives are taken over an arbitrary affine parameter. Substituting Eq. (3) in the Euler-Lagrange equations and parameterizing the ray trajectory in terms of the azimuthal angle *φ* we obtain the first integral of motion


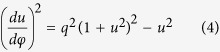


where *u* = *a/r* is the inverse radial coordinate, and *q* = *a*/*b* is the inverse impact parameter. The first integral Eq. (4) can be used to generate the complete phase space of the SPP trajectories in the structured waveguide as shown in Fig. 2(a). A single saddle point (phonon sphere) is observed at *u* = 1 (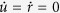) separating the phase space into two distinct domains for GSPPs approaching from infinity. In the first domain, all in-falling rays (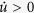) reach a point of closest approach and are then deflected back into infinity, while in the second domains all rays collapse into the central singularity. The actual ray trajectories can be obtained from Eq. (4) which has an explicit solution for in-falling rays in the form:


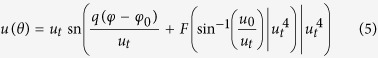


where *φ*_0_ is the angle of incidence, *u*_0_ = *q* sin(*φ*_0_) is the initial position, *F* is the elliptic integral of the first kind, and sn is the Jacobi elliptic function. The solution depends on the external turning point, which is the position of closest approach: 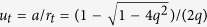. Clearly, for in-falling rays a turning point exists only if *q* ≤ 1/2 or for impact parameters that are larger than a critical value *b*≥*b*_c_ = 2*a*. Otherwise the rays will be captured within the spatial domain below the photon sphere *r* ≤ *a*, as shown in Fig. 2(b,c). Accordingly, our system can be described with a total capture cross-length of *σ*_c_ = 2*b*_c_ = 4*a*, indicating that any GSPP wave that approaches the sliver microsphere within such a spatial domain will be captured and ultimately absorbed. This is also evident by considering the deflection angle for in-falling rays with *b*≥*b*_*c*_ which can be obtained from the inverse of Eq. (5) as:


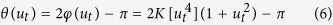


where *K* is the complete elliptical integral of the first kind. For incident GSPP rays that pass far from the photon sphere (*b* ≫ *b*_*c*_), the deflection angle diminishes as *θ* → π*a*^2^/*b*^2^, while for impact parameters close to the critical value (*b* ≈*b*_*c*_), the deflection angle experiences a logarithmic singularity 
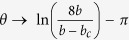
. This critical phenomenon is depicted in Fig. 2(b) and clearly demonstrates that the proposed SMDM waveguide represent a dynamic attractor for GSPP rays. The photon sphere radius *a* of the attractor can be tuned either by varying the radius of the microsphere *R* or the frequency *ω* according to Eq. (2). A particular example is depicted in Fig. 2 (d).

### Adiabatic focusing and energy enhancement

**Figure 3:**
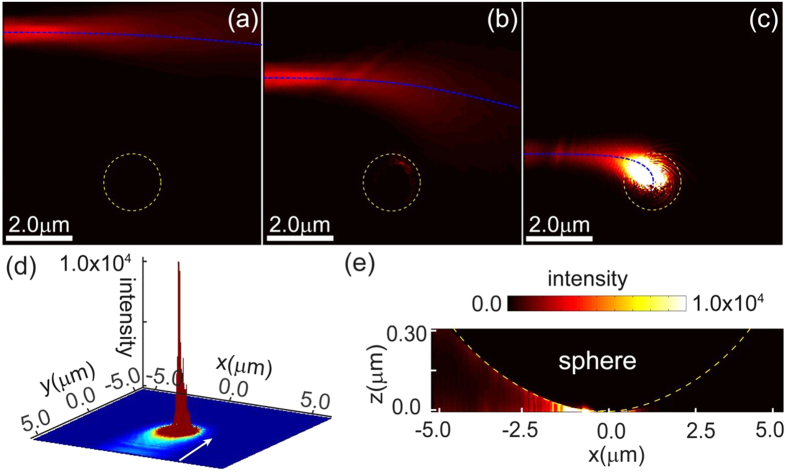
(**a**–**c**) Full wave simulations of GSPP deflection and capture in the structured metal-dielectric metal (SMDM) waveguide. A comparison between the analytical theory Eq. 5 (shown with dashed lined) and simulation results for incident GSPP beams with impact parameters (**a**) *b* = 5*a*, (**b**) *b* = 3.5*a*, and (**c**) *b* = *a*. In the simulations we have fixed *λ* = 0.785 *μm*, and *R* = 40 *μm* which corresponds to a photon sphere radius of *a* = 1.15 *μm*. The location of the photon sphere is indicated with dashed yellow line. (**d**–**e**) The capture of an incident Gaussian shaped GSPP beam with width *σ* = 4*a* results in (**d**) dramatic field enhancement at the contact point between the microsphere and the bottom metal layer (the attractor central singularity) with (**e**) the energy being concentrated within sub-wavelength in size regions.

The geometrical optics approximation used in the analyses from above assumes a gradual change of the refractive index and may not represent well the SPPs in close proximity to the SMDM central singularity. To validate the geometrical optics results we have performed full wave simulations using commercial FDTD software (Lumerical Solutions,Inc.). Figure 3(a–c) shows the simulation results for GSPPs incident on the embedded microsphere with different impact parameters. These results are consistent with the predicted by Eq. (5) ray trajectories and demonstrate both the increase in the deflection angle with decreasing impact factor Fig. 3(a,b), and the capture of the incident GSPP beam for *b* < *b*_c_ = 2*a*, see Fig. 3(c). The trapping of the incident waves at the central attractor results in dramatic increase in the local electric field intensity which reaches values surpassing by a factor of 10^4^ that of the incident, as shown in Fig. 3(d). Furthermore, a cross cut through the microsphere, presented in Fig. 3(e), revels that the energy density is trapped and concentrated within a nano-sized spatial domain in close proximity of the point of contact between the microsphere and the bottom metal layer of the SMDM waveguide.

**Figure 4:**
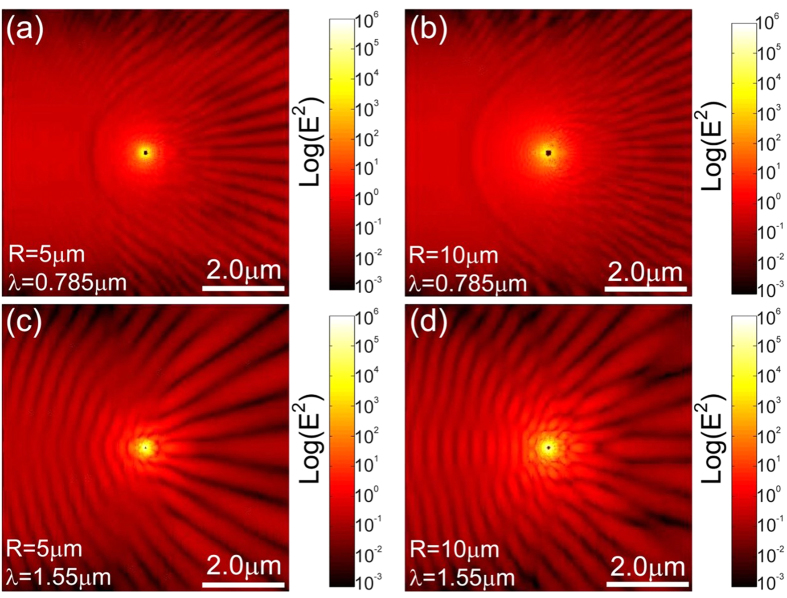
GSPP trapping under various illumination wavelengths and microsphere sizes. The simulation patterns validate the predicted trends for the local field enhancement increases with the incident frequency and microsphere size.

According to the analytical theory the proposed SMDM waveguide acts as an attractor for GSPPs across a broad frequency range. The intensity enhancement at the center of the attractor is proportional to the capture cross-length *σ*_c_ and is expected to increase with decreasing ratio *n*″/*n*′ between the imaginary and real part of the effective index of the GSPP. Considering intermediate frequencies *ω*_*p*_ ≫ *ω* ≫ *ω*_*τ*_, where 
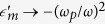
 and 
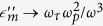
, it follows from Eq. (2) that the index ratio scales as *n*″/*n*′ ≈ (*s*/*a*)^2^ ≈ *ω*_τ_/*ω*. Hence, further increase in the field enhancement can be expected by operating at high frequencies and using larger microspheres. The effects of the microsphere radius and wavelength of illumination are demonstrated in Fig. 4. In the numerical calculations we simulate the three-dimensional structured waveguide as per Fig. 1(a) and have depicted the local field profile for surface cross-section parallel and in close proximity to the bottom metal layer. Our simulations show omnidirectional trapping and ultimately absorption of the incident SPP beam with capture cross section which increases with increasing of the microsphere radius. The field intensity enhancement also increases with decreasing wavelength. These result validate the results obtained based on the geometric optics approximation, by demonstrating adiabatic compression and trapping of GSPPs and exemplifying the two main strategies for improving the energy localization through increase in the microsphere radius and working at high frequencies. The extremely high energy concentration at the center of the attractor can be used to enhance linear and nonlinear effects provided the dielectric material forming the core of the SMDM waveguide is optically active. Such effects will be limited by the GSPP absorption which increases when approaching the central singularity. Furthermore, close to the point of contact with the dielectric gap under 1 nm, non-local effects are expected to further limit the field enhancement^59^,^60^,^61^.

## Conclusions

In this work, we have proposed and investigated a simple and experimental feasible scheme to realize omnidirectional attractors of gap surface plasmon polaritons (GSPP). The proposed structured metal-dielectric-metal (SMDM) waveguide does not require a complicated nanofabrication technology, and provides a natural change in the waveguide thickness according to inserted microsphere surface profile. This results in a centrally symmetric inverse square dependence of the GSPP effective refractive index as function of the distance from the point of contact. The effective index provides for the formation of a broad frequency band optical attractor, i.e. a GSPP black hole, which adiabatically deflects and traps impinging GSPP modes within nano-size spatial domains. The resulting energy localization at the center of the attractor can be used to enhance non-linear optical process such as high-harmonic generation and Raman scattering and may be useful in studies of quantum electrodynamics (QED) effects due to the GSPP trapping.

## Additional Information

**How to cite this article**: Sheng, C. *et al.* Omnidirectional optical attractor in structured gap-surface plasmon waveguide. *Sci. Rep.* **6**, 23514; doi: 10.1038/srep23514 (2016).
